# Natriuretic Peptide Clearance Receptor (NPR-C) Pathway as a Novel Therapeutic Target in Obesity-Related Heart Failure With Preserved Ejection Fraction (HFpEF)

**DOI:** 10.3389/fphys.2021.674254

**Published:** 2021-05-21

**Authors:** Emmanuel Eroume A. Egom

**Affiliations:** ^1^Institut du Savoir Montfort, Hôpital Montfort, University of Ottawa, Ottawa, ON, Canada; ^2^Laboratory of Endocrinology and Radioisotopes, Institute of Medical Research and Medicinal Plants Studies, Yaoundé, Cameroon

**Keywords:** obesity, heart failure with preserved ejection fraction, NPR-C, natriuretic peptide receptor C, adipose tissue, heart failure, co-morbiditie, natriuretic peptides

## Abstract

Heart failure (HF) with preserved ejection fraction (HFpEF) is a major public health problem with cases projected to double over the next two decades. There are currently no US Food and Drug Administration–approved therapies for the health-related outcomes of HFpEF. However, considering the high prevalence of this heterogeneous syndrome, a directed therapy for HFpEF is one the greatest unmet needs in cardiovascular medicine. Additionally, there is currently a lack of mechanistic understanding about the pathobiology of HFpEF. The phenotyping of HFpEF patients into pathobiological homogenous groups may not only be the first step in understanding the molecular mechanism but may also enable the development of novel targeted therapies. As obesity is one of the most common comorbidities found in HFpEF patients and is associated with many cardiovascular effects, it is a viable candidate for phenotyping. Large outcome trials and registries reveal that being obese is one of the strongest independent risk factors for developing HFpEF and that this excess risk may not be explained by traditional cardiovascular risk factors. Recently, there has been increased interest in the intertissue communication between adipose tissue and the heart. Evidence suggests that the natriuretic peptide clearance receptor (NPR-C) pathway may play a role in the development and pathobiology of obesity-related HFpEF. Therefore, therapeutic manipulations of the NPR-C pathway may represent a new pharmacological strategy in the context of underlying molecular mechanisms.

## Introduction

Heart failure (HF) with preserved ejection fraction (HFpEF) is a major public health epidemic with an economic impact that is at least as great as that of HF with reduced ejection fraction (HFrEF) (Upadhya and Kitzman, 2017; Seferovic et al., 2019; Sweeney et al., 2020). HFpEF is common and is becoming increasingly more common (1% growth in cases per year) because of its association with aging and comorbidities (Owan et al., 2006; Sweeney et al., 2020). Despite the high prevalence, there are currently no US Food and Drug Administration–approved therapies for the health-related outcomes of HFpEF, likely due to the marked heterogeneity of the HFpEF syndrome (Oh et al., 2019; Seferovic et al., 2019; Kirkman et al., 2020; Sweeney et al., 2020). Therefore, HFpEF management remains one of the greatest unmet needs in cardiovascular medicine.

The phenotyping of HFpEF patients into pathobiological homogenous groups has been recently suggested for the development of targeted therapies (Obokata et al., 2017; Kirkman et al., 2020). There are distinct phenotypes within this heterogeneous syndrome. Obesity has gained attention as one potential phenotype of HFpEF (Kirkman et al., 2020). Indeed, obesity is highly prevalent in patients with HFpEF as it is estimated that more than 80% of HFpEF patients are either overweight or obese (Shah et al., 2016; Obokata et al., 2017). Elevated body mass index (BMI) is one of the strongest independent risk factors for developing HFpEF, and this excess risk may not be explained by traditional cardiovascular risk factors (Ndumele et al., 2016; Obokata et al., 2017). Although obesity may be associated with other HFpEF comorbidities, recent data suggest that obesity-related HFpEF may represent not only a clinically relevant pathobiological mechanism but also a distinct phenotype within the broad spectrum of HFpEF (obesity-HFpEF phenotype) (Clerico et al., 2018). In addition, obesity may result in the induction of a systemic inflammatory state, which is believed to promote HFpEF progression (Ather et al., 2012; Paulus and Tschöpe, 2013).

There has recently been an increased interest in the intertissue communication between adipose tissue and the heart (Paulus and Tschöpe, 2013; Shah et al., 2016; Obokata et al., 2017; Clerico et al., 2018). Although volume overload may contribute to the development of HFpEF, recent data suggest that the metabolic, endocrine, and natriuretic peptide clearance receptor (NPR-C) signal transduction may play a crucial role in the pathobiology of the obesity-HFpEF phenotype (Matsukawa et al., 1999; Nakatsuji et al., 2010; Bordicchia et al., 2016; Oh et al., 2019). To support this hypothesis, several experimental studies have demonstrated a strong relationship between increased adiposity, NPR-C signaling, arterial hypertension, dyslipidemia, and inflammation and insulin resistance; which, in the long term, may result in impairment of endothelial, diastolic, systolic, arterial, and skeletal muscle functions (Paulus and Tschöpe, 2013; Shah et al., 2016; Obokata et al., 2017; Clerico et al., 2018). This review therefore summarizes the current understanding of the obesity-HFpEF phenotype, focusing on comorbidities and their impact on NPR-C signaling, as well as discussing how “crosstalk” exists between the heart and the adipose tissue within the context of NPR-C pathway.

## Search Methods

For this narrative review, pertinent studies were retrieved from seven electronic databases (PubMed, Google Scholar, Cochrane Library, Web of Science, Science Citation Index, EMBASE, and Elsevier) using common keywords applied in the field of obesity, diabetes, metabolic syndrome, HFpEF, and NPR-C. The author also examined the complete list of relevant references in recent publications in English (both human and studies) on the topics investigated. Given the design of this article as a narrative review, no formal criteria for study selection or quality assessment were applied.

## Natriuretic Peptide Clearance Receptor

**FIGURE 1:**
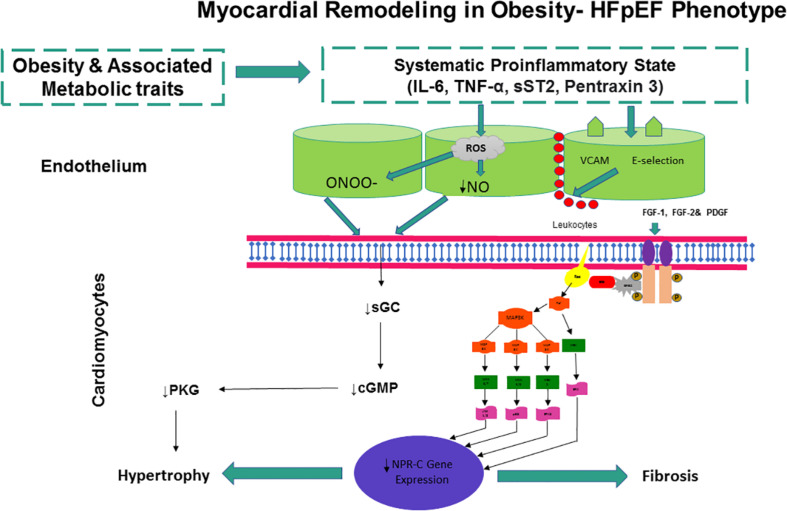
The NPR-C pathway. NPs bind to NPR-C, which is also considered a clearance receptor responsible for receptor-mediated NP degradation. Recent evidence suggests that NPR-C may be coupled to inhibition of adenylyl cyclase (AC) through G_*i*_ protein or activation of phospholipase C (PLC) through G_*i*_ protein. Inhibition of PLC activity may result in decreased production of diacylglycerol (DAG) and inositol triphosphate (IP_3_). NPR-C activation may also have some antiproliferative properties.

Evidence suggests that most of the physiological effects of natriuretic peptides (NPs), including the B-type (BNP), may be mediated through attachment to three distinct NP receptors A, B, and C (NPR-A, NPR-B, and NPR-C) (Egom, 2015; Egom et al., 2015a). The binding of NPs to NPR-A and NPR-B may result in the activation of the guanylyl cyclase (GC) enzyme and subsequent elevation of intracellular cyclic guanosine monophosphate (cGMP), which appears to mediate most of the physiological effects of NPs (Egom, 2015). NPR-C, which is bound by all NPs with similar affinity, does not contain a GC domain and was originally classified as a “clearance receptor” with no signal transduction function (Egom, 2019; Egom et al., 2019). Although the exact contribution of the NPR-C clearance pathway to the NP concentration in the plasma remains unclear, both the NPR-C and neprilysin pathways may contribute to the degradation of NPs (Potter, 2011). Although still commonly called a clearance receptor (Egom, 2015), evidence suggests that NPR-C may be coupled to a pertussis toxin–sensitive inhibitory G protein (G_*i*_) and may reduce adenylyl cyclase activity and intracellular cAMP levels (Figure 1; Katada and Ui, 1982; Zhou and Murthy, 2003).

The relationship between the NP system and adipose tissue is well established. Circulating BNP levels appear to be lower in obese patients than in their normal-weight counterparts. This is even more evident in HFpEF, whereby obese HFpEF patients typically have lower circulating BNP levels than their normal-weight counterparts (Daniels et al., 2006; Obokata et al., 2017; Oh et al., 2019). This complex inverse relationship between circulating BNP and BMI is often termed the “natriuretic handicap” and has been observed in both healthy individuals and patients with HFpEF (Clerico et al., 2018; Nishimura et al., 2018). The molecular mechanisms of the “natriuretic handicap” may be partly related to the differential expression of NPR-C, leading to enhanced degradation of BNP in adipose tissue (Gentili et al., 2017).

Adipose tissue is the second largest expression site of NPR-C after the kidney, and the NPR-C signaling pathway within adipose tissue is the main target for endogenous agonists (Sarzani et al., 2017). Impaired activation of NPR-C signaling has been described in several pathological processes including obesity, inflammation, insulin resistance, fibrosis, ischemia, oxidative stress, remodeling, and arterial and pulmonary hypertension (Hobbs et al., 2004; Egom et al., 2017a,c, 2019; Sarzani et al., 2017; Egom, 2019). The human body may respond to these pathological processes through up-regulation of the NPR-C (a known protective pathway) gene expression as an adaptation mechanism to maintain homeostasis (Gower et al., 2006; Egom et al., 2017a,c, 2019; Sarzani et al., 2017; Egom, 2019). Kuhn et al. (2004) have shown that the mRNA expression levels of the NPR-C receptor were increased in human failing hearts and that the reversal of cardiomyocyte hypertrophy during left ventricular assist device support was accompanied by normalization of the NPR-C mRNA levels. To that end, enhanced NPR-C mRNA levels in various cardiometabolic disorders may represent a compensatory response to low NPR-C activity with the goal of reestablishing cardiometabolic function.

## Obesity-HFpEF Phenotype

The obesity-HFpEF phenotype is growing increasingly more common, with more than 1.8 million patients in the United States currently diagnosed with this clinical entity (Kitzman and Shah, 2016). The obesity-HFpEF phenotype may be considered as a disease of postmenopausal women (Tromp et al., 2019). Among postmenopausal women, HFpEF comprises nearly 90% of incident HF cases (Upadhya and Kitzman, 2017). These women are more likely to have hypertension with significant hypertrophy than age-matched men, as well as higher abdominal fat mass accumulation compared with premenopausal women (Agabiti-Rosei and Muiesan, 2002; Garaulet et al., 2002). The mechanisms that link hypertension, cardiac hypertrophy, obesity, and postmenopausal status have not fully been elucidated because of the lack of suitable experimental models (de Andrade et al., 2011). Experimental models are required tools to investigate the underlying molecular mechanisms linking the NPR-C pathway, obesity-associated cardiometabolic traits, and HFpEF and to explore the use of potential pharmacological agents in this specific phenotype (Valero-Muñoz et al., 2017; Oh et al., 2019). However, until recently, there were no experimental models that could accurately mimic the cardiometabolic changes typically seen in human obesity-related HFpEF (Valero-Muñoz et al., 2017; Oh et al., 2019). Thus, the unavailability of such models may have partly contributed to the current lack of understanding of the molecular mechanisms underlying obesity-HFpEF phenotype.

de Andrade et al. (2011) investigated the role of the NPR-C pathway in the development of obesity-related hypertension and cardiac hypertrophy in ovariectomized fat-fed experimental models. The authors found that the experimental obese postmenopausal models exhibit cardiac hypertrophy, increased mean blood pressure, and increased visceral fat mass, as well as increased NPR-C gene expression (de Andrade et al., 2011). Interestingly, whereas obesity or postmenopausal status alone did not induce the above alterations, a combination of the two conditions (i.e., obesity and postmenopause) was able to trigger some of the cardiovascular and renal alterations that are typically seen in human obesity-related HFpEF (de Andrade et al., 2011). The increased blood pressure and visceral fat mass were strongly correlated with the up-regulation of NPR-C gene expression (de Andrade et al., 2011). In a similar study, Belo et al. (2008) demonstrated that their overweight and estrogen-deficient experimental models had significantly higher renal and mesenteric adipocyte NPR-C gene expression than their wild-type counterparts. This effect was reversed by estrogen treatment, reinforcing the relationship between estrogen, fat deposit, and the NPR-C pathway (Belo et al., 2008).

Evidence suggests that NPR-C gene expression in human adipose tissue may be greater in obese hypertensive than in obese normotensive individuals (Dessì-Fulgheri et al., 1997). In obese individuals, fasting-induced weight loss may result in a reduction of blood pressure, which is accompanied by a significant down-regulation of adipocyte NPR-C receptor (Dessì-Fulgheri et al., 1999). These observations suggest that the altered NPR-C pathway may play a role, at least in part, in a mechanism that reduces blood pressure in obese individuals.

In summary, an impaired NPR-C pathway may contribute to the susceptibility of postmenopausal women in developing hypertension and plays an essential role in the pathobiology of HFpEF in women. It would be interesting to investigate, at least in this patient cohort, the effects of long-term treatment with estrogen on the cardiovascular outcomes.

### Cardiac Comorbidities Associated With Obesity-HFpEF Phenotype

#### Diastolic Dysfunction

Heart failure with preserved ejection fraction was initially labeled as “diastolic HF” based on hemodynamic studies that demonstrated elevated filling pressures in the absence of a significant increase in ventricular chamber size (Zile et al., 2004). As diastolic dysfunction may be observed in subjects with HFrEF, and patients with diastolic dysfunction may also have some degree of systolic dysfunction, the term “diastolic HF” was abandoned and replaced with HFpEF (Oh et al., 2019). Studies using experimental models have highlighted the relationships between the increased adiposity and metabolic alterations observed in obesity, diastolic dysfunction, and HFpEF (Oh et al., 2019). In order to investigate mechanisms linking NPR-C signaling, diastolic dysfunction, and HFpEF, we used an established model of hypertensive heart disease in which mice are chronically administered angiotensin II (Ang II) (3 mg/kg per day for 3 weeks by miniosmotic pump) (Frohlich et al., 1992; Houser et al., 2012; Mackasey et al., 2018). The administration of Ang II results in an initial compensated hypertrophy phase and HFpEF that ultimately transitions to HFrEF (Frohlich et al., 1992; Houser et al., 2012; Mackasey et al., 2018). Using this protocol, we have found that Ang II caused mice to become hypertensive and hypertrophic with diastolic dysfunction, as expected (Mackasey et al., 2018). The echocardiographic assessments (at 3 weeks) showed that wild-type mice developed ventricular hypertrophy but that systolic function was not yet impaired (no strain analysis by speckle-tracking echocardiography was performed) as ejection fraction (EF) and fractional shortening (FS) were not reduced compared to baseline (Mackasey et al., 2018). In contrast, we found that the same Ang II treatment in NPR-C^–/–^ mice greatly expedited disease progression. Specifically, the mice lacking NPR-C that were treated with Ang II showed a much more severe decline in cardiac function and decompensation into overt HFrEF as indicated by ventricular dilatation and reductions in EF and FS (Mackasey et al., 2018).

Strikingly, the cotreatment of wild-type mice with Ang II and a selective NPR-C agonist (cANF) was able to largely prevent or slow the development of diastolic dysfunction, and cANF cotreatment did not prevent the increase in systolic blood pressure elicited by Ang II (Mackasey et al., 2018). Similarly, the increased fibrosis in NPR-C^–/–^ mice is consistent with data demonstrating the strong antiproliferative and antifibrotic effects of NPs on cardiac fibroblasts (Tsuruda et al., 2002; Horio et al., 2003; Kapoun et al., 2004; Kawakami et al., 2004; Huntley et al., 2010), some of which involve NPR-C–dependent signaling (Huntley et al., 2006). In line with these experimental findings, NPR-C genetic alterations were independently associated with the development of diastolic dysfunction in humans, suggesting a potential critical role of NPR-C signaling in the pathobiology of HFpEF (Pereira et al., 2014).

The development of diastolic dysfunction appeared independent of age, gender, body weight, and systemic hypertension in subjects homozygous for the altered NPR3 genotype with an odds ratio of 1.9, which is similar to that of systemic hypertension, a major modifiable traditional cardiovascular risk factor for diastolic dysfunction (Pereira et al., 2014). The potential underlying mechanisms of the development of diastolic dysfunction in this cohort of individuals may be partially due to alterations in the NPR-C’s cytoplasmic domain within cardiac myocytes contributing to cardiac fibrosis and diastolic dysfunction (Pereira et al., 2014; Egom et al., 2015b, 2017a,b).

Taken together, low NPR-C activity may remove the brake on cardiac hypertrophy, thereby inducing cardiac fibrosis and remodeling and leading to diastolic dysfunction, the major cardiac functional deficit in HFpEF.

#### Atherosclerotic Cardiovascular Disease

##### Susceptibility to Atherosclerotic Cardiovascular Disease

Coronary artery disease (CAD) is common and extensive in patients with HFpEF, with a prevalence approaching 40–50% (Shah, 2010; Hwang et al., 2014; Mohammed et al., 2015). In addition, the presence of CAD may predict incident HFpEF and cardiovascular death, especially sudden death (Shah, 2010; Hwang et al., 2014; Mohammed et al., 2015).

Evidence suggests that NPR-C may play a role in the pathogenesis of CAD. Hu et al. (2016) performed a multicenter genome-wide association study in 200 individuals from a Shandong cohort, a pathway-based candidate gene association from a Shanghai cohort (293 CAD/293 controls), and replication studies in additional 3,363 CAD patients and 3,148 controls. They identified new susceptibility loci of *NPR-C* that are specifically associated with CAD (Hu et al., 2016). Interestingly, multivariate logistical regression analyses revealed that the association between these single-nucleotide polymorphisms (SNPs) and CAD remained significant even after adjustment for the conventional atherosclerotic risk factors (age, gender, smoking, hypertension, diabetes, and dyslipidemia), suggesting that the NPR-C gene SNPs contribute to CAD susceptibility (Hu et al., 2016). The molecular mechanisms underlying the association between NPR-C gene polymorphism and CAD have not yet been fully elucidated.

##### Pathobiology of Atherosclerotic Cardiovascular Disease

Natriuretic peptide clearance receptor is expressed in the vasculature and may be involved in cellular proliferation, migration, and vascular remodeling. NPR-C is thus relevant to the study of atherosclerotic cardiovascular disease because vascular cell proliferation and migration are central to the pathophysiology of this inflammatory-based condition. Casco et al. (2002) investigated the pattern of expression of NPR-C in human coronary arteries with various degrees of atherosclerotic lesions and found NPR-C in the intimal and the inner medial layers. Similarly, Naruko et al. (2005) immunohistochemically studied the expression of NPR-C during the post–percutaneous coronary intervention (PCI) healing process and demonstrated that NPR-C was strongly expressed in neointimal vascular smooth muscle cells from 1 to 9 months after PCI. Gene expression and histopathology analyses of coronary artery atheromatous lesions further demonstrated that NPR-C expression may be highest in the intima and inner media layers (Wei et al., 1994; Casco et al., 2002; Naruko et al., 2005). In these studies, as well as in other studies, the expression of NPR-C appeared to positively correlate with the severity of atherosclerotic cardiovascular disease (Furuya et al., 1995; Rollín et al., 2005; Scotland et al., 2005). Furthermore, Zayed et al. (2016) evaluated NPR-C expression by immunohistochemistry in carotid endarterectomy specimens isolated from 18 patients and found significant NPR-C expression in the intima of advanced carotid artery plaques, with an expression pattern correlating to the features of plaque vulnerability. These observations suggest that NPR-C may serve as a potential biomarker for plaque vulnerability and progression in patients with atherosclerotic cardiovascular disease (Zayed et al., 2016).

##### Periprocedural NPR-C Responses

The restoration of coronary blood flow during ischemic myocardium is important for limiting the injury caused by acute myocardial infarction and salvaging cardiac function. However, reperfusion may exert detrimental effects by extending myocardial necrosis and cardiac dysfunction beyond what was achieved by the ischemic insult itself [ischemia/reperfusion (I/R) injury] (Hobbs et al., 2004).

Evidence suggests that the activation of NPR-C signaling may contribute to the regulation of coronary blood flow. NPR-C signaling may also represent a protective mechanism against I/R injury by reducing infarct size and maintaining coronary perfusion pressure and left ventricular developed pressure at preischemic levels (Hobbs et al., 2004). In an experimental heart model, Hobbs et al. (2004) demonstrated that the selective NPR-C agonist cANF^4–23^ elicits the potent relaxation of coronary arteries as well as offers a protective mechanism against I/R injury with suppression of both infarct size and myocardial dysfunction. Furthermore, the administration of cANF^4–23^ during the reperfusion period alone may also protect against I/R injury, suggesting that the activation of NPR-C signaling may prove beneficial in patients presenting with an acute coronary ischemic event (Hobbs et al., 2004).

This NPR-C–induced protective effect may be enhanced in the setting of nitric oxide (NO) synthase inhibition (Hobbs et al., 2004). In fact, evidence suggests that endothelial NO synthase (eNOS) may regulate expression of NPR-C mRNA. Del Ry et al. (2020) investigated the NP system expression in whole blood obtained from normal-weight and obese adolescents and found that individuals with reduced endothelial function may have significantly higher expression level of NPR-C mRNAs. Using experimental models lacking eNOS, Yuan et al. (2010) investigated the effects of eNOS expression on the regulation of NPR-C gene expression. NPR-C mRNA levels were greater in the heart and kidney of the experimental models lacking eNOS compared to the wild-type counterpart (Yuan et al., 2010). Therefore, there may be complementary protective roles for the NPR-C and NO signaling pathways in the cardiovascular system, whereby the loss of one system may be compensated for with the up-regulation of the alternative signal transduction pathway. These complementary roles may be of particular significance for atherosclerotic cardiovascular disorders as they seem to be characterized by loss of the NO pathway (Hobbs et al., 2004). Under such circumstances, the influence of the NPR-C pathway may be heightened. Furthermore, pharmacological agents mimicking the biological activity of the NPR-C pathway may prove to be an important new strategy to treat these disorders.

##### Postprocedural NPR-C Responses

Maintaining the patency and integrity of the coronary arteries after successful restoration of coronary blood flow is of paramount importance. In patients undergoing PCI, reocclusion of the coronary artery often occurs within 6 months (Khambata et al., 2011; Ñato et al., 2018; Neumann et al., 2019a,b; Sousa-Uva et al., 2019; Modi et al., 2021). For these patients with reocclusion, their CAD is treated with a bare metal stent or drug-eluting stent (DES), which acts as a platform for new tissue growth (Khambata et al., 2011; Sousa-Uva et al., 2019). Current DESs (which are now the predominant implanted stents) release antiproliferative drugs that inhibit arterial smooth muscle cell proliferation, which is the most predominant cause of restenosis (Khambata et al., 2011; Ñato et al., 2018; Neumann et al., 2019a,b; Sousa-Uva et al., 2019; Modi et al., 2021). However, these agents may also inhibit endothelial cell proliferation (Matter et al., 2006; Khambata et al., 2011; Ñato et al., 2018; Neumann et al., 2019a,b; Sousa-Uva et al., 2019; Modi et al., 2021), which may increase the thrombogenicity of the stent surface. The antiproliferative effects of NPR-C signaling on vascular cells may therefore provide a promising treatment strategy of restenosis.

The beneficial effects of NPR-C agonists may also be applied to patients undergoing coronary artery bypass grafting (CABG). In patients undergoing CABG, the saphenous vein graft (SVG) is still commonly used as graft material; however, the 1-, 5-, and 10-year postoperative SVG patency rates may be 93, 74, and 41%, respectively (Gao et al., 2018). Furthermore, studies have shown that up to 12% of SVGs occlude within the first 6 months following CABG, with 3.4% occluding as early as 2–3 weeks principally due to thrombosis (Gao et al., 2018). Abnormal hyperplasia of the neointima severely affects a further 10% of grafts within 1 year (1–12 months after CABG) (Gaudino et al., 2017; McKavanagh et al., 2017). Here again, NPR-C signaling manipulation may be of therapeutic benefit. Indeed, NPR-C agonists were shown to be beneficial following balloon angioplasty and vein grafting in experimental models (Khambata et al., 2011).

#### Epicardial Fat

Compared to non-obese HFpEF patients, obese HFpEF patients may display increased epicardial fat thickness (Clerico et al., 2018). Clinical studies have shown that the amount of epicardial adipose tissue (EAT) may be associated with the presence, progression, or severity of CAD (Mahabadi et al., 2013; Nakanishi et al., 2014). Although the underlying pathophysiological mechanisms of EAT in CAD progression are not completely understood, recent evidence suggests that low levels of the EAT NPR-C expression may lead to dysregulation of the epicardial fat surrounding the myocardium, which in turn may contribute, at least in part, to the progression of CAD (Moreno-Santos et al., 2019). Moreno-Santos et al. (2019) investigated the relationship between the expression and signaling of EAT NPR-C and the progression of CAD in humans in a cohort of individuals with angiographically normal coronary arteries, stable CAD, and acute coronary syndrome (ACS). The authors showed that patients with ACS have lower expression of EAT NPR-C at both the protein and mRNA levels compared to patients with stable CAD or angiographically normal coronary arteries (Finck and Kelly, 2006; Díez, 2017; Moreno-Santos et al., 2019). Additionally, the authors showed that patients with ACS have reduced activation of p38 mitogen–activated protein kinase (p38 MAPK); lower expression of EAT uncoupling protein 1 (UCP1), which may in turn lead to reduced thermogenic capacity; and lower expression of peroxisome proliferator-activated receptor γ coactivator α, which may in turn lead to mitochondrial dysfunction.

Moreno-Santos et al. (2019) also found an inverse relationship between the mRNA levels of EAT NPR-C and the severity of CAD. Individuals with 3-vessel disease had lower EAT NPR-C mRNA levels compared with those with 1- or 2-vessel disease and no significant CAD (Moreno-Santos et al., 2019). Multivariate logistic regression models demonstrated significant associations of EAT NPR-C gene expression, EAT PGC1α mRNA levels, and the presence of ACS (Moreno-Santos et al., 2019).

Although it may be premature to make any final conclusions about cause and effect, some observations support the notion that low NPR-C levels may promote plaque vulnerability/instability and progression in patients with atherosclerotic cardiovascular disease (Hobbs et al., 2004; Zayed et al., 2016; Egom et al., 2019). One approach to determine a gene’s function is to observe how a cell or a whole organism behaves when the gene of interest is absent or non-functional. As mentioned previously and as we will discuss further in subsequent sections, low NPR-C activity may remove the brake on cardiac hypertrophy, induce cardiac fibrosis and remodeling, and increase susceptibility to develop arrhythmias and pulmonary hypertension (Hobbs et al., 2004; Egom et al., 2015b, 2019; Jansen et al., 2018, 2019; Mackasey et al., 2018; Egom, 2019). On the other hand, enhanced NPR-C activity may protect against IR injury, reverse cardiac hypertrophy and fibrosis, and improve endothelial function (Hobbs et al., 2004; Egom et al., 2015b, 2019; Jansen et al., 2018, 2019; Mackasey et al., 2018; Egom, 2019).

#### Atrial Fibrillation

Atrial fibrillation (AF) is highly prevalent and commonly occurs in the setting of HFpEF and hypertension (Nattel, 2002; Dobrev and Nattel, 2010), conditions that are characterized by excessive activation of Ang II signaling (Mudd and Kass, 2008). HFpEF and AF may be inextricably linked, both to each other and to adverse cardiovascular outcomes (Chamberlain et al., 2011; Vermond et al., 2015; Upadhya and Kitzman, 2017). The interrelationships between AF and HFpEF remain complex and poorly understood, yet the number of patients with AF and HFpEF continues to increase worldwide (Al-Khatib et al., 2020). Thus, there is a need for experimental work that will provide insight into the mechanisms of the intersection between AF and HFpEF.

We have recently used a well-established model of hypertensive heart disease that leads to cardiac hypertrophy and HFpEF by chronically treating mice with Ang II (Mackasey et al., 2018). Consistent with prior studies (Wakisaka et al., 2007; Swaminathan et al., 2011; Fukui et al., 2013), we demonstrated that chronic Ang II may cause atrial enlargement, electrical and structural remodeling (fibrosis) of the atrial myocardium, and increased susceptibility to AF (Mackasey et al., 2018). In order to investigate the potential role of NPR-C signaling in the interrelationships between AF and HFpEF, we performed experiments in which we treated NPR-C^–/–^ mice (and NPR-C^+/+^ littermates) with saline or Ang II (Mackasey et al., 2018). As mentioned previously, echocardiography assessments demonstrated that NPR-C^–/–^ mice responded much more severely to Ang II than wild-type mice. The NPR-C^–/–^ mice displayed a rapid transition into HFrEF, as demonstrated by ventricular dilatation and reductions in EF and FS, whereas systolic function was not yet impaired in wild-type mice (no strain analysis by speckle tracking echocardiography was performed) (Mackasey et al., 2018). Furthermore, Ang II treatment resulted in significantly more atrial enlargement in NPR-C^–/–^ mice compared to NPR-C^+/+^ littermates (Mackasey et al., 2018). Intracardiac electrophysiology experiments demonstrated that Ang II treatment resulted in a greater susceptibility to AF in NPR-C^–/–^ mice than in NPR-C^+/+^ littermates (Mackasey et al., 2018). Strikingly, cotreatment of wild-type mice with Ang II and cANF prevented atrial electrical and structural dysfunction (Mackasey et al., 2018).

We also demonstrated that the increased susceptibility to AF in NPR-C^–/–^ mice may be caused primarily by enhancing fibrosis in the atria, suggesting that NPs may act upon NPR-C in cardiac fibroblasts to regulate extracellular matrix deposition (Egom et al., 2015b). Consistently, the activation of the NPR-C pathway may have potent antifibrotic and antiproliferative effects on fibroblasts in the heart (Calvieri et al., 2012; Egom, 2019). To investigate the effects of Ang II on AF susceptibility and atrial function, we used *in vivo* electrophysiology, patch clamping, high-resolution optical mapping, and molecular biology on wild-type and NPR-C^–/–^ mice (Jansen et al., 2019). While Ang II increased susceptibility to AF in wild-type mice, these effects were exacerbated in Ang II–treated NPR-C^–/–^ mice (Jansen et al., 2019). Ang II also enhanced fibrosis in both atria in wild-type mice, whereas Ang II–treated NPR-C^–/–^ mice exhibited substantially higher fibrosis burden throughout the atria. Cotreating wild-type mice with Ang II and cANF dose-dependently reduced AF susceptibility by preventing most of the Ang II–induced atrial myocardial abnormalities (Jansen et al., 2019).

Taken together, these findings strongly implicate a potential signaling role for NPR-C in the pathobiology of the complex interrelations between AF and HFpEF.

### Metabolic Traits Associated With Obesity-HFpEF Phenotype

Non-cardiac comorbidities, including but not limited to diabetes and arterial hypertension, are highly prevalent in patients with the obesity-HFpEF phenotype (Upadhya and Kitzman, 2017).

Our previous analysis of mice lacking NPR-C showed that these mice had a lean phenotype with a significantly reduced fat mass (Bordicchia et al., 2012; Egom et al., 2019). Mice lacking NPR-C appeared to have markedly smaller white and brown adipose tissue depots but higher expression of thermogenic genes (such as Ucp1) and other features of brown adipocytes (Bordicchia et al., 2012). The ability of humans to prevent body fat accumulation may be linked to their ability to expand the number and activity of brown adipocytes within white fat depots (Bordicchia et al., 2012). As the mice lacking NPR-C appear to have increased brown adipocytes in their white fat depots, they tend to resist diet-induced obesity and retain insulin sensitivity (Kovacova et al., 2016).

Sarzani et al. (2004) tested the association between the NPR-C A/C(-55)A polymorphism in 787 untreated male participants in the 1994–1995 follow-up examination of the Olivetti Heart Study in Naples. The authors found that individuals carrying the A/C(-55)A *NPRC* genotype had a significantly lower BMI and waist circumference, as well as a significantly lower rate of overweight and obesity at the 20-year follow-up observation (Sarzani et al., 2004). Interestingly, the authors did not find any association between either Blood pressure (BP) or fasting serum insulin concentration and the *NPRC* gene polymorphism (Sarzani et al., 2004).

Obese individuals have higher *NPR-C* gene expression in the subcutaneous abdominal adipose tissue than their lean counterparts (Kovacova et al., 2016). NPR-C is expressed most abundantly in white adipose tissue and more on visceral than subcutaneous adipose tissue (Nakatsuji et al., 2010). Del Ry et al. (2020) analyzed NPR-C expression in whole blood obtained from normal-weight and obese adolescents and found significant associations of circulating insulin, hemoglobin A_1c_ (HbA_1c_) levels, and NPR-C expression, suggesting that hyperinsulinism and altered glucose metabolism may contribute, at least in part, to the elevated NPR-C expression in individuals with high BMI. There is a gradual and progressive increase of *NPR-C* transcripts in adipose tissue when an individual is transitioning from normal glucose tolerance to type 2 diabetes mellitus (T2DM) (Kovacova et al., 2016). Although individuals with normal glucose tolerance may have the same skeletal muscle NPR-C protein content regardless of their BMI, there may be an up-regulation of skeletal muscle NPR-C as glucose tolerance deteriorates in individuals with impaired glucose tolerance or T2DM (Coué et al., 2015).

In addition, hyperinsulinemic states, as found in patients with impaired glucose tolerance/insulin resistance due to metabolic syndrome or diabetes, may up-regulate subcutaneous NPR-C gene expression in a glucose-dependent manner (Nakatsuji et al., 2010). Kovacova et al. (2016) demonstrated that 12 weeks of treatment with pioglitazone significantly lowers levels of *NPRC* mRNA and improves insulin sensitivity in patients with T2DM or metabolic syndrome. Skeletal muscle NPR-C has also been shown to be positively related to fasting blood glucose, insulin, and HbA_1c_, further suggesting that a functional NPR-C pathway is required for insulin sensitivity and blood glucose control (Coué et al., 2015). In a study by Christoffersen et al. (2006), diabetes and impaired glucose metabolism were demonstrated to confer increased NPR-C gene expression in the heart, suggesting a relationship between impaired NPR-C signaling, glucose dysmetabolism, and associated cardiac function.

Although it may be premature to make any final conclusions about cause and effect, the above findings suggest that the NPR-C pathway may be dysregulated in the context of metabolic disorders such as obesity, insulin resistance, and T2DM (Coué et al., 2015). The observed up-regulation of NPR-C mRNAs in these patients with these metabolic disorders may represent a compensatory mechanism to maintain or reestablish cardiometabolic health. The NPR-C pathway may thus represent a novel therapeutic target in cardiometabolic disorders, including but not limited to obesity and insulin resistance, in addition to HFpEF.

## Obesity Paradox

Even though obesity is a strong risk factor for HF in the general population, the “obesity paradox” refers to the fact that obese patients with established HF tend to have better long-term prognosis than non-obese patients (Janovska et al., 2020). Patients with the obesity-HFpEF phenotype have better outcomes than their non-obese counterparts. In a cohort of 150 patients hospitalized with HFpEF, higher BMI values were associated with lower mortality (Stavrakis et al., 2013). Consistently, a U-shaped relationship between BMI and mortality has been reported for HFpEF (Kapoor and Heidenreich, 2010; Padwal et al., 2014; Ohori et al., 2021).

Although the association between obesity and HFpEF is well known, the pathophysiology of weight-related changes on the outcome of patients with HFpEF is still a matter of debate. Nishikido et al. (2019) investigated whether a change in BMI is associated with either prognosis or frequency of hospitalizations in patients who were hospitalized for decompensated HF. The authors found that a lowered BMI may be a significant predictive factor for the frequency of hospitalizations and increased mortality (Nishikido et al., 2019). Similarly, other studies have shown that increased body fat mass, but not appendicular skeletal muscle mass, corresponds to a lower risk of short-term cardiac events in HF patients (Thomas et al., 2019; Ohori et al., 2021). Every 5-unit increase in BMI corresponds to a 10% reduction in mortality (Fonarow et al., 2007).

Unfortunately, most obesity paradox studies used BMI cut points to classify subjects as normal, overweight, or obese (Egom et al., 2018). However, using BMI as a measure of true body fat content has been strongly criticized by some authors who argue that elevated BMI may overestimate the amount of body fat in subjects with greater muscle mass (and thus indicating a more favorable health status) or underestimate it in older individuals because of the loss of muscle mass related to aging (and thus indicating a worse health status) (Kragelund and Omland, 2005; Egom et al., 2018). The measurement of BMI in conjunction with other anthropometric indices such as percentage of waist circumference, body fat, and waist/hip ratio may thus not only be more accurate, but also provide additional prognostic value (Egom et al., 2018).

Although the potential underlying mechanisms of the obesity paradox have been extensively studied (Hamzeh et al., 2017; Thomas et al., 2019; Ohori et al., 2021), little attention has been given to the possible pathophysiological role of NPR-C. Crandall et al. (1989) studied the effects of weight reduction on NPR-C gene expression in an experimental obesity-associated HFpEF model. A cohort of patients who were subjected to a 15-week period of caloric restriction had, on average, a 40% reduction in body weight. These patients also had a significant decrease in NPR-C mRNA and ultimately had worse outcomes (Crandall et al., 1989). Consistently, Crandall et al. (1989) found that obesity-associated cardiac abnormalities persisted even after the normalization of body fat as the patients transitioned from an obese to lean condition, likely due to persistent low activity of NPR-C signaling.

Some evidence suggests that the prognostic impact of increased body fat mass may also be lost systematically as the disease progresses, and HF severity may overcome percent body fat in the prediction of short-term cardiovascular events (Ohori et al., 2021). Indeed, while fat mass may be associated with better survival in patients with advanced HF, cardiac cachexia (non-intentional weight loss) may be a stronger independent predictor for mortality (Janovska et al., 2020). Janovska et al. (2020) investigated the role of the NPR-C pathway in the development of cachexia in patient with advanced HF as well as its potential impact on the EAT–myocardium environment. High levels of mitochondrial phospholipid cardiolipin in various tissues have been causally linked to cachexia. A negative correlation was observed between NPR-C gene expression levels and the mitochondrial phospholipid cardiolipin levels in EAT, supporting the potential role of NPR-C in cardiolipin−induced changes of EAT metabolism in cachexia (Janovska et al., 2020). The authors also found that NPR-C gene expression was twofold to threefold lower in cachectic patients than in their body weight–stable counterparts (Janovska et al., 2020). Interestingly, NPR-C gene expression correlated positively with body weight change, BMI, and daily dose of both β−blockers and angiotensin−converting enzyme inhibitors or angiotensin receptor blockers (Janovska et al., 2020). These conventional HF agents represent the cornerstone of effective HF therapy and should be titrated to the maximally tolerated doses. HF patients with a functional or enhanced NPR-C pathway may be more likely to tolerate higher doses of the neurohumoral inhibitors (Janovska et al., 2020).

The activation of the sympathetic nervous system is one of the compensatory mechanisms mounted by the body to maintain cardiac output in individuals with HF. Evidence suggests that an augmented activation of the sympathetic system may reduce the levels of NPR-C in a time- and dose-dependent manner by decreasing the transcriptional rate of the NPR-C gene (Kishimoto et al., 1994). The catecholamine-induced decrease in NPR-C density may be antagonized by carvedilol (Kishimoto et al., 1994). As obese patients with HF have higher NPR-C expression levels, they may have better tolerance for β-blockers at higher doses and should theoretically have improved outcomes (Litwin, 2008). The rational use of β-blockers in HF for improving the prognosis of the disease may also be supported by this observation (Farré et al., 2015).

The above findings suggest that enhancing the NPR-C pathway may represent an attractive therapeutic strategy to reduce body wasting, increase the ability to tolerate higher HF therapeutic doses, and improve HF outcomes.

## Conclusion

**FIGURE 2:**
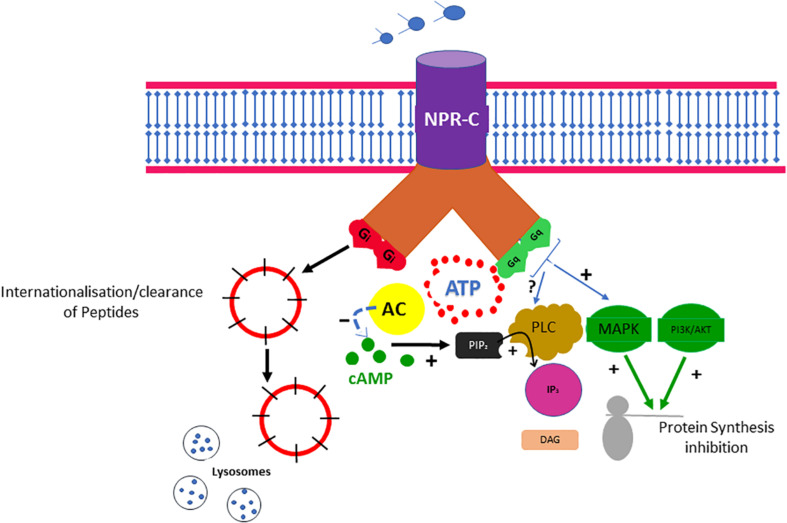
Obesity and associated metabolic traits drive myocardial dysfunction and remodeling in obesity-HFPEF phenotype. Obesity and associated metabolic traits induce a systemic proinflammatory state characterized by high plasma levels of interleukin 6 (IL-6), soluble ST2 (sST2), tumor necrosis factor α (TNF-α), and pentraxin 3. Coronary microvascular endothelial cells produce vascular cell adhesion molecule (VCAM), E-selectin, and reactive oxygen species (ROS). The production of ROS results in reduced NO bioavailability and peroxynitrite (ONOO^–^) production, both of which may lower soluble guanylate cyclase (sGC) activity in adjacent cardiomyocytes. Lower sGC activity results in decreased cGMP concentration and reduced protein kinase G (PKG) activity. Low PKG activity may trigger a cascade of events leading to cardiomyocyte hypertrophy. E-selectin and VCAM expression in endothelial cells may favor migration into the subendothelium of immune cells, which may release cytokines and growth factors including the fibroblast growth factors (FGF-1 and FGF-2) and platelet-derived growth factor (PDGF). FGF-1, FGF-2, and PDGF activate the membrane tyrosine kinase receptors, which then trigger a full range of intracellular Ras-Raf–mitogen-activated protein kinase (MAPK)/extracellular signal–regulated kinase-MAPK signaling transduction pathways, leading to a down-regulation of NPR-C gene expression. Low NPR-C activity may remove the brake on cardiomyocyte hypertrophy, thereby inducing cardiac fibrosis and remodeling, leading to diastolic dysfunction, the major cardiac functional deficit in HFPEF.

Natriuretic peptide clearance receptor signaling may be central to the pathobiology of HFpEF in general and particularly to the obesity-HFpEF phenotype (as illustrated in Figure 2). Addressing the lack of effective available HFpEF therapies remains a priority given the rising demographics of obese, diabetic, hypertensive, and aging populations across the globe. Ultimately, HFpEF and the obesity-HFpEF phenotype are multisystem disorders, and pharmacological agents that alleviate both metabolic and cardiac dysfunction are most likely to provide the greatest clinical benefit. As NPR-C signal transduction may be a point of convergence for all upstream stimuli, from mechanical stretch to endocrine or paracrine mediators, targeting the NPR-C pathway would represent the most promising means of treating the obesity-HFpEF phenotype.

## Author Contributions

The author confirms being the sole contributor of this work and has approved it for publication.

## Conflict of Interest

The author declares that the research was conducted in the absence of any commercial or financial relationships that could be construed as a potential conflict of interest.
